# Beyond Single Nucleotide Polymorphisms: *CYP3A5^∗^3^∗^6^∗^7* Composite and *ABCB1* Haplotype Associations to Tacrolimus Pharmacokinetics in Black and White Renal Transplant Recipients

**DOI:** 10.3389/fgene.2020.00889

**Published:** 2020-08-11

**Authors:** Daniel A. Brazeau, Kristopher Attwood, Calvin J. Meaney, Gregory E. Wilding, Joseph D. Consiglio, Shirley S. Chang, Aijaz Gundroo, Rocco C. Venuto, Louise Cooper, Kathleen M. Tornatore

**Affiliations:** ^1^Department of Pharmacy Practice, Administration and Research, School of Pharmacy, Marshall University, Huntington, WV, United States; ^2^Department of Biostatistics, School of Public Health and Health Professions, University at Buffalo, Buffalo, NY, United States; ^3^Immunosuppressive Pharmacology Research Program, Translational Pharmacology Research Core, NYS Center of Excellence in Bioinformatics and Life Sciences, Buffalo, NY, United States; ^4^School of Pharmacy and Pharmaceutical Sciences, Buffalo, NY, United States; ^5^Jacobs School of Medicine and Biomedical Sciences, University at Buffalo, Buffalo, NY, United States; ^6^Erie County Medical Center, Buffalo, NY, United States

**Keywords:** renal transplantation, immunosuppression, CYP3A5 genotypes, tacrolimus, race, tacrolimus pharmacokinetics, pharmacogenomics, ABCB1 haplotypes

## Abstract

Interpatient variability in tacrolimus pharmacokinetics is attributed to metabolism by cytochrome P-450 3A5 (CYP3A5) isoenzymes and membrane transport by P-glycoprotein. Interpatient pharmacokinetic variability has been associated with genotypic variants for both *CYP3A5* or *ABCB1*. Tacrolimus pharmacokinetics was investigated in 65 stable Black and Caucasian post-renal transplant patients by assessing the effects of multiple alleles in both *CYP3A5* and *ABCB1.* A metabolic composite based upon the *CYP3A5* polymorphisms: *^∗^3*(rs776746), *^∗^6*(10264272), *and ^∗^7(41303343*), each independently responsible for loss of protein expression was used to classify patients as extensive, intermediate and poor metabolizers. In addition, the role of *ABCB1* on tacrolimus pharmacokinetics was assessed using haplotype analysis encompassing the single nucleotide polymorphisms: *1236C* > *T (rs1128503), 2677G* > *T/A(rs2032582), and 3435C* > *T(rs1045642)*. Finally, a combined analysis using both *CYP3A5* and *ABCB1* polymorphisms was developed to assess their inter-related influence on tacrolimus pharmacokinetics. Extensive metabolizers identified as homozygous wild type at all three *CYP3A5* loci were found in 7 Blacks and required twice the tacrolimus dose (5.6 ± 1.6 mg) compared to Poor metabolizers [2.5 ± 1.1 mg (P < 0.001)]; who were primarily Whites. These extensive metabolizers had 2-fold faster clearance (*P* < 0.001) with 50% lower AUC^∗^ (*P* < 0.001) than Poor metabolizers. No differences in C_12 *h*_ were found due to therapeutic drug monitoring. The majority of blacks (81%) were classified as either Extensive or Intermediate Metabolizers requiring higher tacrolimus doses to accommodate the more rapid clearance. Blacks who were homozygous for one or more loss of function SNPS were associated with lower tacrolimus doses and slower clearance. These values are comparable to Whites, 82% of who were in the Poor metabolic composite group. The *ABCB1* haplotype analysis detected significant associations of the wildtype *1236T-2677T-3435T* haplotype to tacrolimus dose (*P* = 0.03), CL (*P* = 0.023), CL/LBW (*P* = 0.022), and AUC^∗^ (*P* = 0.078). Finally, analysis combining *CYP3A5* and *ABCB1* genotypes indicated that the presence of the *ABCB1* 3435 T allele significantly reduced tacrolimus clearance for all three CPY3A5 metabolic composite groups. Genotypic associations of tacrolimus pharmacokinetics can be improved by using the novel composite *CYP3A5^∗^3^∗^4^∗^5 and ABCB1* haplotypes. Consideration of multiple alleles using *CYP3A5* metabolic composites and drug transporter ABCB1 haplotypes provides a more comprehensive appraisal of genetic factors contributing to interpatient variability in tacrolimus pharmacokinetics among Whites and Blacks.

## Introduction

The combination of tacrolimus and mycophenolic acid is the mainstay of maintenance immunosuppressive regimens to prevent renal allograft rejection (Hart et al., 2017; Jouve et al., 2018). Tacrolimus exhibits variable pharmacokinetics and clinical response, necessitating the use of therapeutic drug monitoring (TDM) (Schiff et al., 2007; de Jonge et al., 2009; Shuker et al., 2015, 2016b; Vanhove et al., 2016). However, trough tacrolimus concentration versus effect relationships for clinical responses and adverse drug effects are not well defined in sub-populations stratified by sex or race (Bouamar et al., 2013; Vanhove et al., 2016). Tacrolimus pharmacokinetic and pharmacodynamic variability is attributed, in part to variation in both cytochrome P-450 3A5 isoenzymes and P-glycoprotein (P-gp) (Fredericks et al., 2006; Wang et al., 2006; Staatz et al., 2010a).

The duration of chronic renal allograft survival in Blacks is significantly shorter compared to other races receiving similar immunosuppression (Young and Gaston, 2000, 2002, 2005; Young and Kew, 2005; Eckhoff et al., 2007; Fan et al., 2010; Andrews et al., 2016). Contributing factors include socioeconomics, genomic variants, medication adherence, pharmacokinetic and pharmacodynamic variability, donor-recipient mismatches, time on dialysis and racial variation in immunodynamic responses (Young and Gaston, 2005; Young and Kew, 2005; Eckhoff et al., 2007; Page et al., 2012). Interestingly, Blacks require higher tacrolimus doses compared to Whites to achieve similar allograft outcomes (Neylan, 1998; Young and Gaston, 2005; Vadivel et al., 2007). Tacrolimus bioavailability is reduced in healthy Blacks (Fitzsimmons et al., 1998; Mancinelli et al., 2001). Though not examined here, sex is another recognized factor influencing the activity of CYP3A4/5 isoenzymes and P-glycoprotein (Cummins et al., 2002; Scandlyn et al., 2008; Hu and Zhao, 2010; Soldin et al., 2011; Momper et al., 2017).

Pharmacogenomic testing has been incorporated into clinical studies of calcineurin inhibitors (Barbarino et al., 2013; Hesselink et al., 2014; Bruckmueller et al., 2015; Andrews et al., 2016; Tang et al., 2016; Meng et al., 2018) and may be responsible for the differences among races. For example, the wild-type variant, *CYP3A5^∗^1*, is common in Blacks as compared to *CYP3A5^∗^3* (rs776746) the major variant in Whites which is associated with loss of protein expression. This variant (*CYP3A5^∗^3*) contributes to interpatient tacrolimus pharmacokinetic variability as reflected in dose-normalized trough, area under the concentration vs. time curve (AUC) and clearance (MacPhee et al., 2004; Dai et al., 2006; Thervet et al., 2010; de Jonge et al., 2012; Andrews et al., 2016; Oetting et al., 2016; Shuker et al., 2016a). However, in Blacks, other *CYP3A5* variants including *CYP3A5^∗^6* (rs10264272) and *CYP3A5^∗^7* (rs41303343), are associated with loss of function, and may also contribute to interracial variability in tacrolimus pharmacokinetics (Birdwell et al., 2015; Oetting et al., 2016; Sanghavi et al., 2017). Recent studies in Black recipients with *CYP3A5^∗^1* alleles require higher daily tacrolimus doses than Whites with the variant *CYP3A5^∗^3*, or Blacks exhibiting variants *CYP3A5^∗^3*, *CYP3A5^∗^6*, and/or *CYP3A5^∗^7* to achieve comparable troughs (Birdwell et al., 2015; Oetting et al., 2016; Sanghavi et al., 2017). Our group has recently reported the *CYP3A5^∗^3^∗^6^∗^7* metabolic composite to provide a comprehensive phenotypic representation (Campagne et al., 2018).

Pharmacokinetic estimates in these studies were limited to trough concentrations which has limitations in describing accurate tacrolimus exposures. Variable correlations between tacrolimus troughs and area under the concentration curve (AUC) have been reported contributing to interpatient pharmacokinetic variability (Schiff et al., 2007; Staatz et al., 2010b). Therefore, an alternative study design that uses intensive sampling to characterize tacrolimus AUC or drug exposure in African American and Caucasian recipients is needed to guide further development of dosing strategies in relation to wild-type *CYP3A5^∗^1* and variant *CYP3A5^∗^3^∗^6^∗^7* genotypes (Birdwell et al., 2015).

In contrast to the relationship of tacrolimus pharmacokinetics to CYP3A5^∗^1/^∗^3 variants, the relationship of the ATP binding cassette gene subfamily B member 1*(ABCB1)* variants, as surrogate markers for P-gp has an unclear relationship to calcineurin inhibitor pharmacokinetics and pharmacodynamics (Staatz et al., 2010b; Knops et al., 2013; Hesselink et al., 2014). P-glycoprotein serves as an adenosine triphosphate (ATP)-dependent efflux pump for substrates, such as calcineurin inhibitors(CNI), resulting in reduction of systemic exposure and lower intracellular drug accumulation (Huang et al., 2010; Cascorbi, 2011; Hodges et al., 2011; Barbarino et al., 2013). Extensive P-gp tissue distribution, reinforces its functional contribution in the development of adverse effects (Haufroid et al., 2006; Provenzani et al., 2011; Knops et al., 2013; Hesselink et al., 2014; Stefanovic et al., 2015; Venuto et al., 2015). Alterations in P-gp expression or function have been attributed to genetic polymorphisms, race, sex, environment, or endogenous inhibitors (Cattaneo et al., 2009; Huang et al., 2010; Hodges et al., 2011; Barbarino et al., 2013; Hesselink et al., 2014). Reports regarding the influence of common ABCB1 single nucleotide polymorphisms (SNPs): *1236C* > *T (rs1128503), 2677G* > *T/A (rs2032582), and 3435C* > *T (rs1045642)* have focused on tacrolimus pharmacokinetics or renal pharmacodynamics including acute rejection and nephrotoxicity (Staatz et al., 2010b; Hesselink et al., 2014). Conflicting reports have examined individual SNPs, an approach that may not include the effect of multiple *ABCB1* polymorphisms and their interrelationship to selected tacrolimus pharmacokinetics or associated adverse effects (Staatz et al., 2010b; Knops et al., 2013). These commonly evaluated *ABCB1* SNPs are inherited as a haplotype with distinct racial frequencies (Kim et al., 2001; Kimchi-Sarfaty et al., 2007a; Hodges et al., 2011). Due to linkage disequilibrium, the *1236T-2677T-3435T (TTT)* haplotype is the most prevalent variant, and is associated with significant reductions in P-gp activity compared to wild type (Kimchi-Sarfaty et al., 2007b). This haplotype variant is postulated to decrease P-gp activity and subsequently impact systemic tacrolimus exposure and increase intracellular drug exposure with the potential for increased adverse effects (Staatz et al., 2010b; Picard and Marquet, 2011; Hesselink et al., 2014). Different frequencies of *ABCB1* SNPs and haplotypes between Blacks and Whites have been described and should be considered in pharmacogenomic analysis (Kim et al., 2001; Keskitalo et al., 2008; Woodahl et al., 2008; Kassogue et al., 2013). The inclusion of *ABCB1* haplotypes may provide more insightful associations to pharmacokinetic and adverse effects phenotypes during tacrolimus immunosuppression (Kimchi-Sarfaty et al., 2007a; Liu et al., 2008, 2009; Staatz et al., 2010b). Most studies including *ABCB1* SNPs or haplotypes have investigated either tacrolimus dose-normalized troughs or daily doses and acute rejection with no evaluation of the important non-renal adverse effects (Staatz et al., 2010b; Hesselink et al., 2014).

The objectives of this study were to assess: (1) the influence of CYP*3A5* metabolic composite genotypes combining three common loss of function SNPs, *CYP3A5^∗^3* (rs776746), *CYP3A5^∗^6* (rs10264272), and *CYP3A5^∗^7* (rs41303343) on tacrolimus pharmacokinetic phenotypes; (2) to assess tacrolimus pharmacokinetics in association to *ABCB1* haplotypes; and (3) an integrated analysis combining the novel CYP*3A5* metabolic composite and *ABCB1 3435 (rs1045642*) to assess the combined role of the two loci to tacrolimus pharmacokinetic parameters.

## Materials and Methods

### Study Population

Sixty-five (33 Black and 32 White) stable male and female renal transplant recipients receiving tacrolimus (*Prograf)* and mycophenolic acid as enteric-coated mycophenolate sodium (ECMPS; *(Myfortic)* for ≥6 months participated in a 12-h pharmacokinetics-pharmacogenomic study. Patients were recruited by a nephrologist during their transplant clinic visit if they demonstrated clinical stability in renal function, clinical laboratory tests and concurrent disorders. Physical exams, comprehensive metabolic panels including liver and renal function tests, electrolytes, glucose, albumin and protein concentrations with complete blood counts and differentials were used to confirm clinical stability. Tacrolimus doses were adjusted to 4–9 ng/ml troughs based upon time post-transplant and clinical response using a program-specific minimization protocol. ECMPS was dose adjusted based upon clinical response. Estimated glomerular filtration rate (e-GFR) was calculated using the four-factor MDRD equation (Levey et al., 1999). Medication adherence was verified by transplant nurse clinician and medication adherence assessment by transplant pharmacist at enrollment. Ethnicity for two previous generations was verified prior to study.

Inclusion criteria were: (1) ≥6 months post-renal transplant; (2) age 25–70 years; (3) first or second deceased-donor or living allograft recipient; (4) same immunosuppressive doses for ≥7 days; (5) Serum creatinine ≤3.25 mg/dl with no change >0.25 mg/dl during prior 2 visits; (6) leukocyte count ≥ 3000/mm^3^ and hemoglobin ≥8.0 g/dl. Exclusion criteria were: (1) infection or acute rejection within 2 weeks; (2) drugs interfering with tacrolimus or MPA absorption; (3) cytochrome P4503A4/3A5 or P-glycoprotein inhibitors or inducers within 4 weeks; (5) significant medical or psychiatric diseases that would limit participation.

### Study Procedure

This was a cross-sectional, open-label pharmacokinetic-pharmacogenomic study in stable male and female Black and White recipients conducted at the University at Buffalo (UB) Renal Research Center at the Erie County Medical Center (ECMC). The UB Health Sciences Institutional Review Board approved the study (IRB# PHP0599703-4) which was conducted in accordance with the ethical standards for human subjects and the 1964 Helsinki Declaration. Upon enrollment, patients provided written consent after review of the study purpose, risks and benefits.

All patients were at steady-state conditions for both tacrolimus and ECMPS. Patients were enrolled only if they had received the same dose of tacrolimus and ECMPS for ≥7 days prior to study. This was assumed to be sufficient to approach steady-state plasma concentrations. Proton pump inhibiters, H_2_ antagonists and antacids were discontinued at least 36 h prior to study. Patients took immunosuppressives between 5:30 to 6:30 PM prior to study, fasted and abstained from caffeine and alcohol for 12 h prior to study. At 6:00 AM, patients were admitted, vital signs documented and an intravenous angiocatheter inserted. A 0 h sample (∼15 ml) was collected prior to immunosuppressives for drug troughs and laboratory tests (ECMC Clinical Chemistry Laboratory). Oral study medications [(single lot of tacrolimus *(Prograf) and* ECMPS *(Myfortic*)] were administered at 7:00 AM. Patients remained upright throughout the study. Standardized low fat meals were provided after 4 h. Antihypertensives were administered after 1.5 h and non-immunosuppressives after 4 h. Blood samples (7 ml) were collected at 0 h and 1, 2, 3, 4, 6, 8, 10, and 12 h after drug administration. Whole blood samples were aliquoted within 30 minutes and stored at −70°C until analysis.

Blood was collected in cell preparation tubes (CPT- BD Vacationer) with sodium citrate pre-dose for separation of peripheral blood mononuclear cells (PBMCs) according to processing protocol at 25°C. Plasma was aspirated with PBMC harvested; immediately frozen in liquid nitrogen and stored at −70°C until genotype analysis.

### Genetic Analysis

All blood samples provided viable DNA for genotyping and were analyzed at the University of New England Genomics Research Core. Genomic DNA was isolated from 600 μl of PBMCs per manufacturers’ protocol (Wizard^§^ Genomic DNA Purification. Promega Madison, WI). Personnel with no knowledge of clinical data assayed for *CYP3A5* variants, *CYP3A5^∗^3* (rs776746), *CYP3A5^∗^6* (rs10264272), and *CYP3A5^∗^7* (rs41303343) and *ABCB1* SNPs: *1236C* > *T(rs1128503), 2677G* > *T/A (rs2032582), and 3435C* > *T (rs1045642)*. Ten ng of patient genomic DNA was used to characterize each single nucleotide polymorphism (SNPs) using validated *TaqMan* allelic discrimination assays (Thermo Fisher Scientific, Applied Biosystems, Foster City, CA) with Bio-Rad Laboratories CFX96 Real-Time Polymerase Chain Reaction Detection System (Hercules, CA). For each SNP assay, duplicate samples were analyzed. All protocols and sample handling were in accordance with published guidelines. Allele frequencies for all SNPs were confirmed to be in Hardy-Weinberg equilibrium when adjusted for race.

Given the known linkage among all three *ABCB1* SNPs, haplotype analysis was conducted. Haplotype analysis provides greater power to detect potential unknown functional variants than SNPs alone (Venuto et al., 2015). *ABCB1* haplotype estimation was determined using the THESIAS program (Tregouet et al., 2004; Tregouet and Garelle, 2007). THESIAS uses a maximum likelihood algorithm for the simultaneous estimation of haplotype frequencies and their association to tacrolimus pharmacokinetic parameters. Significant associations for tacrolimus pharmacokinetics as phenotypic means with confidence intervals for each haplotype on a single chromosome were reported.

### Metabolic Composite *CYP3A5^∗^3^∗^6^∗^7* Analysis

**FIGURE 1:**
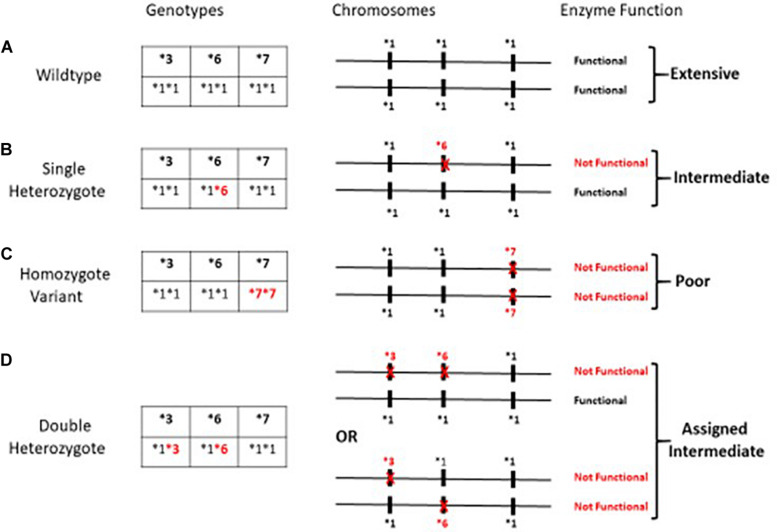
Metabolic Composite scoring algorithm for CYP3A5*3*6*7 SNPs. Metabolic Composite Score for hypothetical patients based upon the combined allelic status from each chromosome is summarized in **(A–D)** above. **(A–D)** illustrates a few of the possible arrangements of the three alleles on the chromosomes and not specific allelic arrangements that have been described to date. **(A)** depicts an Extensive Metabolizer with two completely functional genes. **(C)** depicts individuals who carry a loss of function allele on both chromosomes and are Poor Metabolizers. **(B,D)** represents Intermediate Metabolizers due to at least one loss of function allele (Campagne et al., 2018).

The variants, *CYP3A5^∗^3*, *CYP3A5^∗^6*, and *CYP3A5^∗^7*, all result in loss of protein gene expression (Birdwell et al., 2015). Loss of protein function due to any one of these variants can occur independent of allelic status at the other two loci; thus assessment of any single SNP may be misleading as an indicator of enzyme function. Patients were assigned a metabolic composite designation as described earlier (Campagne et al., 2018) based upon the combined allelic status at all three independent loci (Figure 1). The Extensive Metabolizer phenotype was assigned to individuals with functional genes on both chromosomes. Patients were assigned the Poor Metabolizer phenotype if they were homozygous for the variant allele at any one of the three SNPs and thus CYP3A5 genes on both chromosomes are non-functional (Figure 1C). Patients were designated as Intermediate Metabolizer if they were heterozygous for one loss of function SNP at any of the three loci (Figure 1B). Finally, for patients who were heterozygous at two or more of the SNPs responsible for loss of function, the level of enzyme is dependent upon the arrangement of the variant alleles on each chromosome (Figure 1D). For example, if all of the loss of function SNP’s are located on the same chromosome, this individual would have one “functional” gene similar to the single heterozygote. For this analysis double heterozygotes (Figure 1D) were conservatively assigned as an Intermediate Metabolizer since individuals could either be poor or intermediate metabolizers.

### Combined CYP3A5 Metabolic Composite and ABCB1 and Tacrolimus Pharmacokinetics

To assess the role of both *CYP3A5* and *ABCB1* variants on tacrolimus pharmacokinetics the *CYP3A5* metabolic composite groups and the *ABCB1 3435C* > *T* (rs1045642) were included in a single analysis. The *ABCB1 3435C* > *T* variant was used as a proxy for wildtype *ABCB1* haplotype in this combined analysis since the haplotype assignment algorithm, THESIAS, does not assign individual haplotype scores to individuals. Significant effects of both *CYP3A5* and *ABCB1* variants on tacrolimus pharmacokinetics was assessed using multivariate analysis of variance.

### Assay Methodology for Tacrolimus

Tacrolimus troughs and pharmacokinetic concentrations were analyzed within 24 h at the ECMC Clinical Laboratory using the *ARCHITECT* tacrolimus assay (Abbott, Abbott Park, IL), a chemiluminescent microparticle immunoassay. The lower limit of detection was 0.5 ng/ml and intraday assay variability was <7%. The calibration standard curve ranged from 1 to 30 ng/ml and quality controls (QC) were 3.0, 12.0, and 25 ng/mL (Bio-Rad, Hercules, CA, United States). The interday coefficient of variation (CV) for each QC was <4% and intra-day CV was <5%. Selected troughs and peaks (*N* = 40 samples) were analyzed using a validated LCMSMS assay by a CLIA certified external analytical laboratory and compared to the results generated from the *ARCHITECT* tacrolimus assay with excellent agreement (R^2^ = 0.98). For the tacrolimus LCMSMS assay, the interday and intraday CV were <5% at the low and high concentration QC.

### Pharmacokinetic Analysis

Pharmacokinetic parameters included area under the concentration versus time curve 0 to 12 h (AUC_0__–__12 *h*_), dose-normalized AUC_0__–__12_ (AUC^∗^); 12-h trough (C_12 *h*_) and peak concentration (C_*max*_) with dose normalization and time to peak (T max). Oral clearance of tacrolimus was the ratio of dose to AUC_0__–__12 *h*_. Tacrolimus clearances were adjusted for TBW and LBW. AUC_0__–__12_ was determined by the linear trapezoidal rule using non-compartmental methods (*Phoenix WINNONLIN* Version 6.3. *Pharsight Corp, Mountain View, Calif*). C_12 *h*_, C_*max*_, AUC_0__–__4 *h*_, and AUC_0__–__12_ were dose-normalized to 1 mg dose equivalent.

### Statistical Analysis

All patient demographics and tacrolimus pharmacokinetic parameters were summarized by metabolic composite score for *CYP3A5^∗^3^∗^6^∗^7* using the mean and standard deviation for continuous variables. The potential trend between metabolic composite genotypes and association to tacrolimus pharmacokinetics were evaluated using the two-sided Jonckheere-Terpstra test for trends (Jonckheere, 1983). *Post hoc* pairwise comparisons were made using Holm-Bonferroni adjusted Wilcoxon rank sum tests (*version 9.3, SAS Institute, Cary, NC*). Significant combined effects reflecting composite metabolic *CYP3A5* and *ABCB1* variants on tacrolimus pharmacokinetics was assessed using multivariate analysis of variance (Littell et al., 2006). Significant effects are shown in bold.

## Results

### Patients

Sixty-five recipients completed the study with no statistical differences in age or time post-transplant albumin, liver function tests and hematologic parameters were within normal range for patients with no group differences (Table 1). Mean MPA doses were not different among groups.

**TABLE 1:** Patient demographics clinical characteristics adjusted for CYP3A5*3*6*7 metabolic composite groups.

		CYP3A5*3*6*7 Metabolic Groups				
		Poor	Intermediate	Extensive	Overall	JT Trend *P*-value
	N (%)	35 (53.8)	23 (35.4)	7 (10.8)	65 (100%)	
Age (yrs)	Mean/Std/N	49.8/12.6/35	48.5/11.0/23	45.4/9.0/7	48.9/11.6/65	0.426
Gender	Male	16 (45.7%)	9 (39.1%)	4 (57.1%)	29 (44.6%)	
	Female	19 (54.3%)	14 (60.9%)	3 (42.9%)	36 (55.4%)	0.690
Race	Black	6 (17.1%)	20 (87.0%)	7 (100.0%)	33 (50.8%)	
	White	29 (82.9%)	3 (13.0%)		32 (49.2%)	**<0.001**
Time Post-Transplant (yrs)	Mean/Std/N	2.9/3.0/35	3.4/2.0/23	2.5/1.9/7	3.0/2.6/65	
						***P*-value**
Serum Creatinine (mg/dl)		1.3 (0.3)	1.6 (0.40	1.7 (0.4)	1.4 (0.4)	**0.001**
Estimated Glomerular Filtration Rate ADJ Black Female (ml/min/1.73 m^2^)		58.4 (14.3)	54.0 (17.4)	48.4 (13.1)	55.8 (15.5)	0.237
Glucose (mg/dl)		114.7 (68.8)	122.8 (79.3)	93.0 (20.8)	115.3 (69.2)	0.449
Total White Blood Cells (x10^3^ cells/mm^3^)		5.4 (2.1)	4.9 (1.7)	5.9 (1.9)	5.3 (1.9)	0.361
Platelets (cells x 10^6^)		196.4 (46.5)	195.6 (60.6)	213.0 (45.4)	197.9 (51.3)	0.472
Hemoglobin (g/dl)		12.4 (1.4)	12.2 (1.4)	12.2 (1.2)	12.3 (1.4)	0.870
Body Mass Index (kg/m^2^)		29.8 (5.5)	30.1 (6.9)	31.6 (6.6)	30.1 (6.1)	0.758
Albumin (g/dl)		4.1 (0.3)	4.1 (0.30	4.1 (0.3)	4.1 (0.3)	0.874
Prednisone N(%)		5 (14.3%)	6 (26.1%)	2 (28.6%)	13 (20.0%)	0.457
MPA trough at 12 h (mcg/dl)		3.2 (1.7)	4.1 (2.2)	4.2 (2.9)	3.7 (2.0)	0.305

**P*-values from two-sided Jonckheere–Terpstra Trend (JT) test.*

### *CYP3A5^∗^3^∗^6^∗^7* Genotype Associations With Tacrolimus Pharmacokinetics

As previously described, the CYP3A5 metabolic composite (Campagne et al., 2018; Figure 1), was used to assess the role of multiple genotypes on tacrolimus pharmacokinetics. There were no significant differences among metabolic composite groups for age, gender and time post-transplant as well as clinical measures (Table 1). Frequency differences among metabolic composite groups between races were significant. For the metabolic composite *CYP3A5^∗^3^∗^6^∗^7* frequencies Extensive Metabolizers were identified in 7 Blacks with no Extensive Metabolizers among White patients. Twenty-three patients were genotyped as Intermediate Metabolizers with 20 Blacks and 3 Whites. Poor Metabolizers consisted of 29 Whites and 6 Blacks.

**FIGURE 2:**
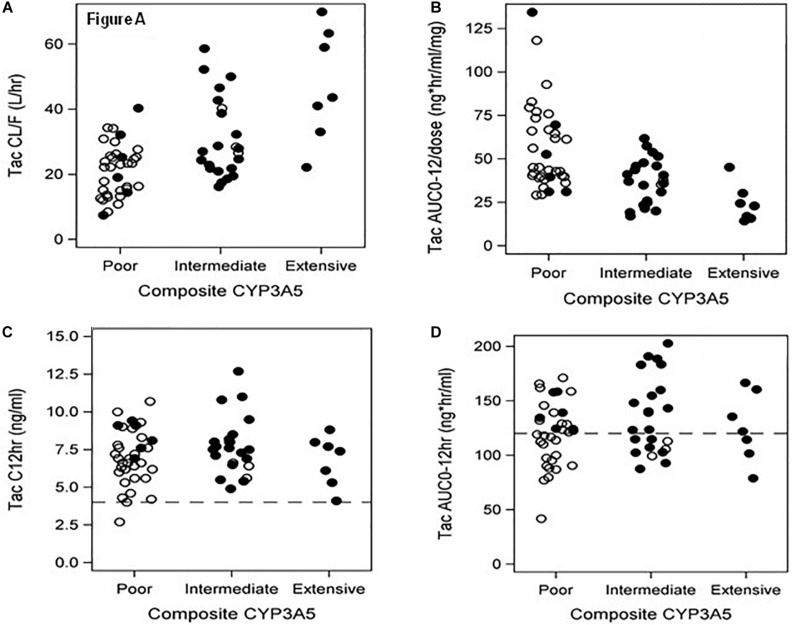
**(A–D)** Metabolic Composite for CYP3A5*3*6*7 and associations to tacrolimus pharmacokinetic parameters – **(A)** represents tacrolimus clearance classified by 3 metabolic composite groups for CYP3A5*3*6*7. The Extensive Metabolizers are all Blacks with more rapid clearance than Poor Metabolizers (*P* < 0.001), who were primarily Whites. **(B)** depicts dose normalized AUC _0–12_ with Poor Metabolizers with twice the dose normalized tacrolimus exposure compared to Extensive Metabolizers (*P* < 0.001); **(C)** presents tacrolimus troughs divided by metabolic composite groups using the target range of >4 ng/ml and <15 ng/ml for our study. No difference was found between these groups. Note that 64 of 65 patients are within the therapeutic trough range. **(D)** depicts AUC_0–12 *h*_ graphs of the metabolic composite groups using the tacrolimus target of >120 and ≤200 ng.hr/ml (Wallemacq et al., 2009). Note that 17/32(53%) of Whites and 10/33(30%) of Blacks had 12-h tacrolimus exposures <120 ng.hr/ml distributed across all groups in spite of the therapeutic troughs **(C)**. OPEN Circle = Whites; CLOSED Circle = Blacks.

Significant associations of tacrolimus pharmacokinetics with *CYP3A5^∗^3^∗^6^∗^7* metabolic composite are summarized in Table 2. Extensive Metabolizers exhibited a 2-fold greater tacrolimus dose (*P* < 0.001) and Dose/TBW (*P* < 0.001) compared to Poor Metabolizers. Although no difference was noted with troughs between the 3 metabolic composite groups (Figure 2C), a 2.5-fold greater dose normalized trough (Cp_12__*hr*_/dose) (*P* = 0.0016) was found in Poor compared to Extensive Metabolizers (Table 2). Tacrolimus clearance (*P* < 0.001) was twice as rapid in Extensive Metabolizers compared to Poor Metabolizers (Figure 2A). The dose-adjusted AUC_0__–__12 *h*_ (*P* < 0.001) was 2 fold higher in Poor than Extensive metabolizers (Figure 2B), though no difference between groups was found with AUC_0__–__12 *h*_ (Figure 2D).

**TABLE 2:** CYP3A5*3*6*7 metabolic composite groups and associations to tacrolimus pharmacokinetics.

	Composite CYP3A5*3*6*7 Metabolic Groups				Group Pairwise Comparison*		
Tacrolimus pharmacokinetic parameters	Poor (1)	Intermediate (2)	Extensive (3)	JT Trend *P*-value	1 vs. 2	1 vs.3	2 vs. 3
	Mean/Std/N	Mean/Std/N	Mean/Std/N				
Study dose (mg)	2.46/1.06/35	4.07/1.57/23	5.64/1.60/7	**<0.001**	**<0.001**	**<0.001**	**0.039**
Study dose/TBW (mg/kg)	0.03/0.02/35	0.05/0.02/23	0.07/0.02/7	**<0.001**	**0.004**	**0.004**	0.062
C_12h_ (ng/ml)	6.99/1.83/35	7.70/1.88/23	6.77/1.67/7	0.550	0.737	0.808	0.808
C_12_/dose (ng/ml/mg)	3.32/1.64/35	2.13/0.81/23	1.31/0.63/7	**<0.001**	**0.005**	**<0.001**	**0.011**
Cmax (ng/ml)	16.89/6.90/35	20.41/8.14/23	20.81/13.48/7	0.129	0.249	1.000	1.000
Cmax/Dose (ng/ml/mg)	7.69/3.33/35	5.49/2.23/23	4.00/2.69/7	**<0.001**	**0.029**	**0.016**	0.155
T max (hr)	1.81/0.81/35	2.17/1.50/23	1.52/0.54/7	0.954	0.729	0.729	0.580
AUC _0–12_ (ng.hr/ml)	119.66/28.73/35	135.75/35.14/23	125.66/31.30/7	0.261	0.515	1.000	1.000
AUC* (ng.hr/ml/mg)	56.03/24.69/35	37.06/12.83/23	24.26/10.80/7	**<0.001**	**0.005**	**0.002**	**0.019**
CL_F (L/hr)	21.14/8.02/35	30.79/12.19/23	47.43/17.30/7	**<0.001**	0.008	**0.002**	**0.019**
CL/LBW (L/hr/kg)	0.38/0.16/35	0.55/0.27/23	0.91/0.40/7	**<0.001**	**0.036**	**0.003**	**0.044**
AUC _0–4_ (ng.hr/ml/mg)	51.20/15.22/35	60.77/20.07/23	56.89/19.24/7	0.153	0.285	1.000	1.000
AUC^∗^_0–4_ hr (ng.hr/ml/mg)	23.77/10.53/35	16.46/6.22/23	11.02/5.24/7	**<0.001**	**0.016**	**0.004**	0.056

*P*-values from two-sided Jonckheere–Terpstra Trend (JT) test. *Post hoc pairwise comparisons using Holm-Bonferroni adjusted Wilcoxon rank sum tests.

### ABCB1 Variants and Associations With Tacrolimus Pharmacokinetic Parameters

**FIGURE 3:**
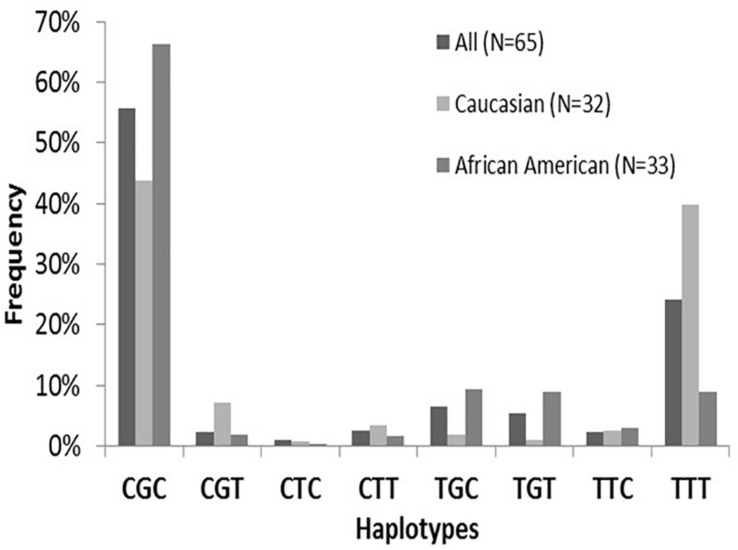
*ABCB1* haplotype frequencies by Race. Note the distributions of ABCB1 wild-type CGC compared to variants. The frequency of the major variant is TTT is depicted with a frequency of 39.8% in Whites compared to 8.9% in Blacks. There were significant overall differences (*p* < 0.001) for haplotype frequencies between Whites and Blacks attributed to CGC and TTT (brackets). This finding is consistent with previously evaluated populations (29–31). No differences in haplotype frequencies are found between sexes. Figure from Venuto et al. (2015).

The *ABCB1* SNPs:*1236C* > *T(rs1128503), 2677G* > *T/A (rs2032582), and 3435C* > *T (rs1045642)* were assessed using validated TaqMan allelic discrimination assays. Hardy-Weinberg equilibrium was confirmed for allele frequencies at each position. Linkage disequilibrium (LD) among the three *ABCB1* SNPs was found to be significant and ranged from 0.89 (*ABCB1* 2677–3435) to 0.72 (*ABCB1* 1236–3435). Estimated *ABCB1* haplotype frequencies (*n* = 65) are summarized in Figure 3 and do not vary significantly compared to previously reported estimated frequencies (Venuto et al., 2015). The most frequent variant haplotype (TTT) displayed significantly different frequencies between Whites and Blacks compared to the wild-type CGC (Venuto et al., 2015).

The significant associations of tacrolimus pharmacokinetic parameters with *ABCB1* variants are summarized in Table 3. A significant association of the TTT variant was found with maximum concentration/dose (Cmax/Dose) (*p* = 0.050) with no sex or race association. With sex-adjusted Thesias analysis, significant associations of TTT haplotypes were found with tacrolimus study dose (*P* = 0.03), oral clearance at steady state (*P* = 0.023), CL/LBW (*P* = 0.022) with lower doses and slower clearances in Whites. With race adjusted Thesias analysis, an association of TTT was noted with AUC_0–4_ (*P* = 0.041) and a trend noted with AUC^∗^ (*p* = 0.078) with lowest exposures found in White males.

**TABLE 3:** *ABCB1* haplotype associations with tacrolimus pharmacokinetics.

Tacrolimus pharmacokinetic parameter	Wild Type Haplotype^*a*^ (CGC) Association to Phenotypic Mean^*b*^ [95% CI]	Variant Haplotype^*a*^ and Phenotypic Mean^*b*^ (95% CI)		*P*-value^*c*^
		Variant haplotype	Phenotypic mean (95% CI)	
Study dose (mg)	2.45 [1.74 – 3.16]*	TTT	1.58 [0.71 – 2.45]*	**0.03**
CL_F (L/hr)	20.4 [14.5 – 26.3]*	TTT	13.99 [7.87 – 20.11]*	**0.023**
Clearance/LBW (L/hr/kg)	0.48 [0.37 – 0.60]*	TTT	0.36 [0.23 – 0.47]*	**0.022**
AUC* (ng.hr/ml/mg)	80.0[68.8 – 91.1]	TTT	92.2 [74.6 – 109.8]	0.078
AUC_0–4_ (ng.hr/ml/mg)	36.59 [30.2 – 42.9]	TTT	43.9 [33.4 – 54.4]	**0.041**
C max/Dose (ng/ml/mg)	3.01[2.42 – 3.59]	TTT	4.06[3.19 – 4.93]	**0.050**

*The phenotypic means of the wildtype haplotype (CGC) vs. phenotypic means for variant haplotype (TTT). ^*a*^Haplotypes displayed at positions 1236-2677-3435. Wild-type is C-G-C. ^*b*^Phenotypic mean estimated from maximum likelihood methods by THESIAS; Phenotype reported as contribution of single haplotype (one chromosome). **^*c*^***P* value represents variant haplotype comparison to wild-type by THESIAS. ^∗^Sex-adjusted analysis; +race-adjusted analysis.*

### Combined Role of *CYP3A5* and *ABCB1* on Tacrolimus Pharmacokinetics

**FIGURE 4:**
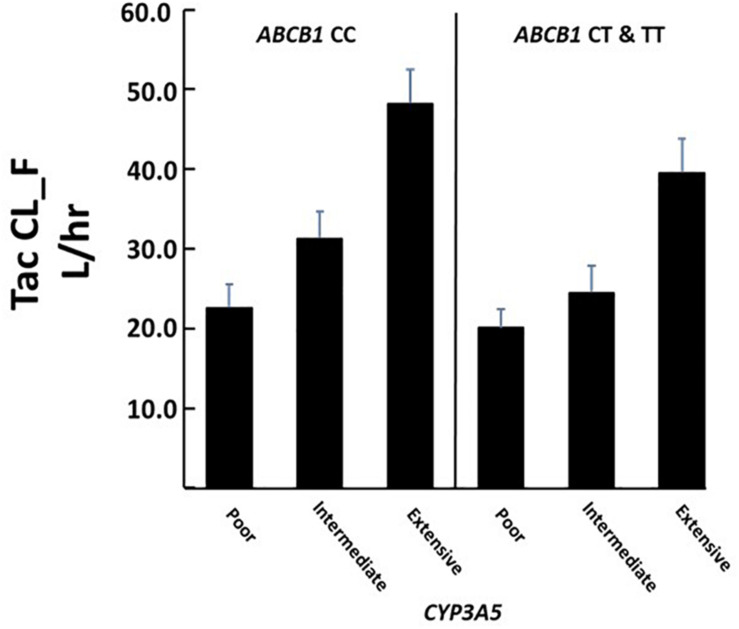
Association of CYP3A5 metabolic composite groups and *ABCB1 T* allele with Tacrolimus clearance. There were significant differences in clearance among metabolic groups (overall *P* < 0.00001) and between individuals carrying at least one *ABCB1 T* allele (*P* = 0.040). Significant effects were determined using multivariate analysis. Corresponding N’s for each group are given in Table 4.

The combined role of both *CYP3A5* and *ABCB1* variants on tacrolimus pharmacokinetics was assessed by including both the *CYP3A5* metabolic composite groups and the *ABCB1 3435C* > *T (rs1045642)* in a single analysis. As observed in the individual CYP3A5 composite analysis above, significant associations of tacrolimus pharmacokinetics with *CYP3A5^∗^3^∗^6^∗^7* metabolic composite were again observed even with the inclusion of *ABCB1* variants (Table 4). Significantly, for apparent clearance (Tac CL_F L_hr) both the *ABCB1* variant and the CYP*3A5* metabolic composite had a significant effect. Across all 3 metabolic composite groups, the presence of an *ABCB1 3435 T* allele significantly reduced tacrolimus clearance beyond that accounted for by CYP3A5 variants alone (Figure 4).

**TABLE 4:** Tacrolimus pharmacokinetics stratified by CYP3A5*3*6*7 metabolic composite groups and *ABCB1 3435* genotypes.

	*ABCB1 3534 CC*			*ABCB1 3534 CT and TT*			Significance	
	*CYP3A5* Poor	*CYP3A5* Intermediate	*CYP3A5* Extensive	*CYP3A5* Poor	*CYP3A5* Intermediate	*CYP3A5* Extensive	*ABCB1 3435*	*CYP3A5* Composite
N	13	10	6	20	10	6		
Study dose (mg)	2.46 (0.36)	4.00 (0.41)	5.67 (0.53)	2.35 (0.29)	3.55 (0.41)	5.08 (0.53)	0.281	**2.2E-08**
Study Dose/TBW (mg/kg)	0.03 (0.01)	0.05 (0.01)	0.07 (0.01)	0.03 (0.00)	0.04 (0.01)	0.06 (0.01)	0.846	**1.3E-05**
C_12*hr*_ (ng/ml)	6.94 (0.50)	7.55 (0.58)	7.40 (0.74)	6.81 (0.41)	8.37 (0.58)	6.58 (0.74)	0.930	0.107
C_12*hr*_/dose (ng/ml/mg)	3.44 (0.37)	2.16 (0.43)	1.43 (0.55)	3.30 (0.30)	2.40 (0.43)	1.57 (0.55)	0.814	**2.3E-04**
Cmax (ng/ml)	15.68 (2.23)	18.62 (2.54)	16.97 (3.29)	16.96 (1.80)	23.33 (2.54)	23.67 (3.29)	0.057	0.099
Cmax/Dose (ng/ml)	7.20 (0.81)	5.21 (0.92)	3.36 (1.19)	8.09 (0.65)	6.61 (0.92)	4.95 (1.19)	0.105	**0.002**
Tmax (hr)	1.94 (0.31)	1.87 (0.35)	1.78 (0.46)	1.72 (0.25)	2.29 (0.35)	2.03 (0.46)	0.628	0.740
AUC* (ng.hr/ml)	116.68 (8.54)	130.75 (9.73)	121.82 (12.57)	117.75 (6.88)	150.30 (9.73)	129.45 (12.57)	0.263	**0.036**
AUC_0–12_ Dose (ng.hr/ml/mg)	56.39 (5.64)	36.76 (6.43)	23.95 (8.30)	56.80 (4.55)	42.94 (6.43)	28.97 (8.30)	0.485	**1.1E-04**
CL_F (L/hr)	22.61 (2.94)	31.30 (3.35)	48.23 (4.32)	20.09 (2.37)	24.49 (3.35)	39.53 (4.32)	**0.040**	**2.8E-07**
CL/LBW (L/hr/kg)	0.37 (0.06)	0.55 (0.07)	0.90 (0.09)	0.39 (0.05)	0.41 (0.07)	0.79 (0.09)	0.218	**7.2E-07**
AUC _0–4 *h*_ (ng.hr/mL/mg)	48.59 (4.75)	57.57 (5.41)	51.85 (6.99)	50.96 (3.83)	68.17 (5.41)	61.47 (6.99)	0.110	**0.032**

*Univariate results from multivariate analysis of variance. Values are Mean (Std).*

## Discussion

P-glycoprotein interacts with gastrointestinal and hepatic cytochrome P450 3A isoenzymes to modulate tacrolimus pharmacokinetics which impacts systemic and cellular drug distribution (Fan et al., 2010; Hesselink et al., 2014; Shuker et al., 2016b). Thus both *ABCB1* and *CYP3A5* variants play an essential role in modulation of intracellular tacrolimus concentrations (Kim et al., 2001; Fredericks et al., 2006; Cattaneo et al., 2009; Hesselink et al., 2014) and overall systematic tacrolimus exposure. Therefore, assessments of the multiple polymorphisms in both genes may assist in understanding tacrolimus pharmacokinetics. We present a pharmacogenetic analysis employing a *CYP3A5^∗^3^∗^6^∗^7* composite and *ABCB1* haplotypes to clinically identify rapid versus poor metabolizers of tacrolimus.

The majority of Blacks required higher daily doses and exhibited more rapid tacrolimus clearance attributed to *CYP3A5* in Extensive and Intermediate metabolic groups compared to Poor Metabolizers. The Clinical Pharmacogenetics Implementation Consortium (CPIC) has provided guidelines to incorporate these individual genotypic variants with tacrolimus dosing to account for inter-patient pharmacokinetic variability (Birdwell et al., 2015). We report the association of tacrolimus pharmacokinetics to the CYP3A5 metabolic composite. Approximately 17% of Blacks were identified as Poor Metabolizers due to the presence of CYP3A5^∗^6/^∗^7 variants. These individuals were similar to White Poor metabolizers whose reduced CYP3A5 function is due primarily to a different allele (*CYP3A5^∗^3*). With increasing admixture among racial groups, the presence of multiple loss of function alleles will warrant the use of composite scoring. Studies investigating troughs as a surrogate marker for drug exposure in Black recipients have demonstrated associations with *CYP3A5^∗^1* alleles with higher dose requirements to achieve therapeutic troughs comparable to *CYP3A5^∗^3^∗^6^∗^7* (Oetting et al., 2016). While limited to troughs, these studies also did not include sex specific analysis or intensive sampling to accurately verify tacrolimus exposure over the 12-h dosing interval achieved during maintenance immunosuppression. Therefore, utility of these genotype-based dosing models may have limitations (Oetting et al., 2016; Sanghavi et al., 2017). Our study reinforces the interpatient variability in tacrolimus exposure with approximately 40% of patients exhibiting AUC_0–12 *h*_ below the recommended therapeutic range in spite of achieving target troughs (Figure 2D).

This is the first prospective study to incorporate *ABCB1* haplotype analysis with a CYP3A5 composite phenotype with intensive tacrolimus pharmacokinetics. Evaluation of *ABCB1* genotypes and in some cases, haplotypes, as indirect markers of cellular P-gp has been used to identify patients at higher risk for calcineurin inhibitor associated adverse effects including nephrotoxicity, gingival hyperplasia and neurotoxicity with tacrolimus dose normalized troughs or daily doses with conflicting results (Yamauchi et al., 2002; Drozdzik et al., 2004; Hauser et al., 2005; Kotrych et al., 2005; De Iudicibus et al., 2008; Garcia et al., 2013; Venuto et al., 2015). The validity of haplotype-based analysis has been widely accepted in association studies of unrelated individuals (Shuker et al., 2016a). Haplotype analyses using maximum likelihood methods combined with Stochastic Expectation-Maximization (SEM) algorithms have recently been used to assess candidate genes with specific phenotypes (Tregouet et al., 2004; Venuto et al., 2015). Haplotypic data provide greater power to detect associations compared to single genotypes especially when analyzed in conjunction with demographic and clinical covariates (Akey et al., 2001; Little et al., 2009). Inclusion of the 3 loci as *ABCB1* haplotypes improves detection probability relative to the pharmacokinetic parameters. The significant associations of TTT haplotypes to tacrolimus pharmacokinetics provides novel and important information that needs to be evaluated in a larger renal transplant population.

Although haplotype association studies provide a comprehensive view of genetic variants, limitations do exist. These limitations reflect small sample sizes, inconsistent use of haplotype analyses, and multiple testing. Due to our sample size, some of the rare haplotypes were poorly represented and may limit our ability to detect significant effects for those very rare haplotypes. Individual *ABCB1* SNPs: *rs1045642 (C3435T)* and *rs203582 (G2677A)* have variable outcomes as pharmacogenomic predictors with different drug substrates; thus, the haplotype approach may improve detection ability for phenotypic differences (Chinn and Kroetz, 2007).

Given the role of both *CYP3A5* and *ABCB1* variants on tacrolimus pharmacokinetics, a combined analysis using both genotypes were examined. While the role of CYP3A5, as determined by the metabolic composite had the largest effect of tacrolimus clearance (Tac CL_F L _hr), the presence of the *ABCB1 3435 T* allele (here used as a proxy for the wildtype haplotype) was significantly associated with reduced tacrolimus clearance for all metabolic composite groups (Figure 4). Fredericks et al. (2006) found a minor effect due to *ABCB1* haplotypes on tacrolimus dose requirements. This effect was lost when the analysis further included patients who were classified as producers or non-producers of *CYP3A5* (based upon the presence of the *CYP3A5^∗^3 allele*). Our significant finding may be due to (1) the inclusion of genotypic data for *CYP3A5^∗^6 and ^∗^7* and both studies had Black patients where these alleles are common; (2) the use of a metabolic composite score; and (3) the comparison of different pharmacokinetic parameters.

The advantages of our study include the prospective enrollment of stable patients at steady state dosing conditions during intensive 12-h pharmacokinetic evaluation that quantitated actual drug exposure reflecting therapeutic drug monitoring of trough concentrations. All patients were enrolled using pre-determined Inclusion and Exclusion criteria which is an important advantage. Multivariate analysis also incorporated common clinical covariates to further identify patients at risk for adverse effects or variability in tacrolimus pharmacokinetics. Another advantage is the inclusion of *ABCB1* haplotype analysis to minimize the limitations of multiple allele test of individual variants and phenotypic endpoints. The use of intensive pharmacokinetic profiles in patients was combined with adherence assessment and tacrolimus concentrations measured in a single CLIA certified drug analysis laboratory provides an important study advantage. Using this approach provides consistency and accuracy for drug concentration analysis to use in determination of comprehensive tacrolimus pharmacokinetic parameters and systematic exposure. Finally, the inclusion of a metabolic composite to represent loss of function due to combinations of *CYP3A5^∗^3^∗^6^∗^7* SNPs further identified factors that influence interpatient variability in tacrolimus pharmacokinetics in the groups. Our statistical model incorporated use of pair-wise group comparisons to substantiate differences in pharmacokinetics and pharmacogenomic associations that may improve our understanding of sub-population differences.

There are some limitations that should be considered from the findings of this report. Our assignment of CYP3A5 metabolic composites for the double heterozygotes is hampered by the fact that the use of individual genetic assays for each SNP do not provide information on the chromosomal arrangement of the variant alleles. Similarly, the use of Thesias to estimate *ABCB1* haplotype phenotypic effects is necessary since chromosomal arrangement of the three *ABCB1* SNPs are not known given the individual genetic assays used. Sequencing could solve this problem but would be prohibitively time consuming and expensive for even this study with this modest sample size.

This study provides support for a more comprehensive inclusion of multiple alleles either by utilizing composite metabolic scores for *CYP3A5* where multiple alleles such as ^∗^3, ^∗^6 and ^∗^7 likely have the loss of function phenotype and with the *ABCB1* haplotype analysis in situations where linkage combines alleles of unknown effects that may impact systemic distribution of tacrolimus. The combining of tacrolimus pharmacokinetics with a more inclusive representation of multiple alleles that reflect proteins that impact drug metabolism and distribution may provide more clinical utility in therapeutic drug monitoring of this immunosuppressive post-transplant.

## Data Availability Statement

The datasets for this article are not publicly available because access to de-identified patient data from this clinical study cannot be provided due to stipulations in the sponsor specific agreement from Astellas Scientific and Medical Affairs, Inc. Requests to access this data can be addressed to the corresponding author.

## Ethics Statement

The studies involving human participants were reviewed and approved by University at Buffalo Health Sciences Institutional Review Board (IRB# PHP0599703-4). The patients/participants provided their written informed consent to participate in this study.

## Author Contributions

DB, RV, GW, and KT were involved in the study design of this project. RV, SC, AG, and KT were involved in recruitment and clinical evaluation of patients. DB, KA, GW, CM, JC, LC, and KT were involved in the data analysis of this project. DB, KA, GW, CM, JC, LC, RV, SC, AG, and KT were involved in writing this manuscript. All authors contributed to the article and approved the submitted version.

## Conflict of Interest

The authors declare that the research was conducted in the absence of any commercial or financial relationships that could be construed as a potential conflict of interest.

## Abbreviations

*ABCB1*: ATP binding cassette gene subfamily B member 1
AUC 0-12: Area under the concentration vs. time curve from 0 to 12 h
AUC^∗^: dose normalized AUC 0 to 12 h
AUC 0–4 hr: Area under the concentration vs. time curve from 0 to 4 h
AUC^∗^ 0–4 hr: dose normalized AUC 0–4 hr
BMI: Body mass index
CNI: Calcineurin inhibitors
*CYP3A5*: Cytochrome P-450 3A5 isoenzymes
*CYP3A5^∗^3^∗^6^∗^7* metabolic composite: genotypic variants *CYP3A5^∗^3*: *CYP3A5^∗^6, CYP3A5^∗^7*C_12 *h*_: 12 h trough concentration
C 2 h: 2 h concentration
C max: Maximum concentration
CLss: steady state oral clearance
ECMPS: enteric Coated Mycophenolate Sodium
MPA: Mycophenolic Acid
N: Number of patients
P-gp: P glycoprotein
LBW: Lean body Weight
SNP: Single Nucleotide Polymorphism
Std: Standard Deviation
T_*max*_: Time to maximum concentration
TBW: Total Body Weight.
